# Engaging community leaders to improve male partner participation in the prevention of mother-to-child transmission of HIV in Dar es Salaam, Tanzania

**DOI:** 10.1371/journal.pone.0207986

**Published:** 2018-12-12

**Authors:** Goodluck Willey Lyatuu, Helga Naburi, Roseline Urrio, Shally Zumba Mwashemele, Sarah Mdingi, Rehema Panga, Happiness Koda, Yusuph Chende, Martha Tsere, Aisa Mhalu, Helen Siril, Irene Andrew Lema, Eric Aris, Aisa Nkya Muya, Maria Rosaria Galanti, Gunnel Biberfeld, Charles Kilewo, Anna Mia Ekström

**Affiliations:** 1 Management and Development for Health, Dar es Salaam, Tanzania; 2 Department of Obstetrics and Gynecology, Muhimbili University of Health and Allied Sciences, Dar es Salaam, Tanzania; 3 Department of Public Health Sciences, Global Health (IHCAR), Karolinska Institutet, Stockholm, Sweden; 4 Department of Pediatrics and Child Health, Muhimbili University of Health and Allied Sciences, Dar es Salaam, Tanzania; 5 Ubungo Municipal Council, Dar es Salaam, Tanzania; 6 Department of Infectious Disease, Karolinska university hospital, Stockholm, Sweden; The Ohio State University, UNITED STATES

## Abstract

**Background:**

Male partner participation improves uptake, retention and outcomes of prevention of mother-to-child transmission of HIV (PMTCT) services. However, in patriarchal settings few men accompany their partners to antenatal care (ANC) and PMTCT services. We explored whether community leaders can improve male partner participation in ANC and PMTCT.

**Methods:**

We integrated initiatives to increase male partner participation in routine ANC care in six health facilities (attending about 4,500 new pregnant women per quarter) in Dar es Salaam, Tanzania in 2015/16. These initiatives were adapted from a best performing health facility, on male partner participation in ANC and PMTCT, referred to as the *“best practice site”*. At the six purposively selected intervention sites, we sensitized and garnered commitment from healthcare providers to provide couple friendly services. We then worked with the providers to sensitize and engage community leaders to integrate and promote male partner participation initiatives in their routine community activities. We assessed change in male partner participation in ANC and PMTCT using the proportion of women testing for HIV together with their partners (i.e. couple HIV testing) by quarter. We used 203 ANC facilities (attending about 31,000 new pregnant women per quarter) in the same area as control sites.

**Results:**

After one year, couple HIV testing in the six intervention sites had tripled from 11.9% at baseline to 36.0% (p<0.001) while there was very little change (from 17.7% to 18.3%) in the 203 control sites (p = 0.07). Statistically significant improvements in couple testing were observed in four of the six intervention sites (6.7% to 19.1%; 9.3% to 74.6%; 46.2% to 95.2%; and 4.7% to 15.1% respectively. p<0.001 for all sites). Two of these four sites, located in the same administrative district as the best practice site, achieved remarkably high couple HIV testing (95.2% and 74.6%). This may be attributable to the greater engagement and active participation of the community leaders in these two sites compared to the other four.

**Conclusion:**

Effective engagement and functional partnerships between healthcare providers and community leaders can contribute to improve male partner participation in ANC and PMTCT services. PMTCT programs should capitalize on community leaders, in addressing low male partner participation in ANC and PMTCT, in order to improve effective uptake, retention and outcomes of HIV prevention and treatment services among pregnant and breastfeeding women, their partners, infants and families.

## Introduction

At the end of 2015, it became clear that the global plan towards elimination of new HIV infections among children in the 21 priority countries in Africa, targeted by the Joint United Nations Program for AIDS Relief (UNAIDS), would not reach its goal [1]. Although the global plan countries achieved 60% reduction in new HIV infections among children from 2009 to 2015, the 110,000 children newly infected with HIV in 2015 was a staggering number at a time when all the tools to achieve elimination of mother-to-child transmission of HIV (eMTCT) were in place [1]. Effective use of lifelong antiretroviral therapy (ART) among pregnant and breastfeeding women is known to reduce the risk of mother-to-child transmission of HIV (MTCT) to less than 5% in breastfeeding and less than 2% in non-breastfeeding populations [2–7]. The World Health Organization (WHO) recommends universal access to and use of lifelong ART in all pregnant and breastfeeding women living with HIV for prevention of mother-to-child transmission of HIV (PMTCT), i.e. the WHO Option B+ [2].

In 2015 the MTCT rate in the 21 Global plan priority countries was estimated to be 8.9%, despite the implementation of the Option B+ in almost all of them [1]. Furthermore, the MTCT rate almost doubled from 4.7% at 6 weeks of age to 8.9% at the end of breastfeeding [1]. Eighty percent of pregnant women living with HIV were reported to have used antiretroviral medicines for PMTCT [1], 10% short of the 90% target. Poor adherence to ART and poor retention in care were highlighted as major challenges limiting the success of PMTCT [1, 8–11]. Barriers to uptake, adherence and retention in PMTCT services that have been reported include: low male partner participation, non-disclosure of HIV status among couples, lack of continued support and counseling on adherence, inadequate knowledge and awareness, HIV-related stigma, competing priorities in everyday life, and other health system related factors [12–19].

Meaningful participation of male partners in ANC and PMTCT care can greatly contribute to address these barriers and improve uptake, adherence, retention and outcomes in PMTCT [20–23]. A systematic review of 34 studies in 11 countries, mostly in sub-Saharan Africa, showed that male partner participation in PMTCT was associated with up to 40% reduction in the risk of MTCT [23]. Pregnant women who attend ANC together with their partners are normally offered the opportunity to receive couple HIV counseling and testing and share their test results with support from health care providers [20]. This allows them to receive HIV prevention education and counseling hence stimulating dialogue on safer sex. Couple HIV testing also enables partners with discordant HIV test results to be linked to ART services for the HIV-positive partner, which is known to reduce the risk of HIV transmission among couples by 96% (so called treatment as prevention) [24]. Furthermore, male partner involvement and couple ANC attendance has been shown to improve knowledge and awareness on pregnancy care, birth preparedness, health facility delivery and childcare among women and their partners [25, 26]. Factors that affect male partner participation in ANC and PMTCT services include: gender roles stereotypes, prevailing perceptions of ANC as a women-only clinic, competing economic demands, lack of awareness, HIV-related stigma, limited physical space at ANC clinics, and non male-friendly environment at ANC clinics [27–35]. Several approaches aimed to improve male partner participation in ANC and PMTCT have been previously investigated, in the past, with varying effectiveness. These approaches include: written and verbal invitation letters to male partners, creating male friendly environments at ANC and providing health talks on male participation during ANC clinic [20, 36–39]. However, few studies have specifically explored interventions targeting male partner participation at first ANC visit, a critical entry point for couple HIV counseling and testing, and PMTCT. We therefore designed and evaluated an intervention to improve male partner participation at first ANC visit, couple HIV testing, and PMTCT through engagement of community leaders.

## Materials and methods

### Setting

We conducted an implementation evaluation study in routine healthcare settings of the Dar es Salaam region, the main commercial city of Tanzania. In 2015/16, when this study was implemented, the region had a projected population of 5.5 million people, 51.3% female, and an annual population growth rate of 5.6% [40, 41]. The region had a total fertility rate of 3.6 per woman and an annual crude birth rate of 37 per 1,000 women [42]. The most recent data indicates that 4.7% of the adult population older than 15 years of age in Dar es Salaam is HIV positive, with a much higher prevalence among women (6.8%) than among men (2.3%) [43]. Among women giving birth in the region, 98% attend ANC at least once during pregnancy and 84% (higher than national average of 63%) deliver in a health facility [44]. In 2015, when the intervention was implemented, the Dar es Salaam region had 258 public and private health facilities providing ANC services [45]. Among these sites, 223 (86%) received technical and financial support for HIV prevention and treatment services from the Management and Development for Health (MDH). MDH is a non-governmental public health organization that has been commissioned by the Tanzania Ministry of Health to provide technical support in the clinical management of HIV services in 2 regions of Tanzania. MDH’s technical support entails capacity building of healthcare providers and health system strengthening in areas of human resource, infrastructure, supply chain, laboratory services and monitoring and evaluation.

### Design

This was a non-randomized implementation evaluation study. The study was embedded in routine health care settings with participating health facilities serving as units of observation and analysis. Six health facilities were purposively selected to be intervention sites and 203 other facilities selected as control sites. The main outcome of interest was the proportion of women who received HIV counseling and testing together with their male partners during their first ANC clinic visit (i.e. couple HIV testing).

The main intervention of this study was based upon lessons from one primary care public health facility in Dar es Salaam region that succeeded to increase male partner participation in ANC and PMTCT from 1% in 2010 to 92% in 2014, herein referred to as the *“best practice site”*. Healthcare providers at this best practice site were motivated to improve male partner participation in ANC and PMTCT services at their site after attending a PMTCT training organized by MDH. Male partner participation at this site was proxy measured by the proportion of pregnant women who attended and tested for HIV together with their partners at first ANC clinic visit.

The six purposefully selected intervention sites, herein referred to as sites A to E, were primary care facilities with similar client volumes (about 300 to 1,200 new pregnant women per quarter) as the best practice site (about 500 new pregnant women per quarter), and low couple HIV testing rates the previous year. Furthermore, the intervention sites were evenly distributed across the three administrative districts of Dar es Salaam region at that time. We purposely selected sites with dedicated healthcare providers who would be willing to put in the extra effort required to make the intervention successful. The main reason for limiting the intervention to six sites was to reduce overall costs by focusing on high quality implementation of the intervention, to assess its feasibility and scalability in other similar settings as well as to understand the feasibility of replicating the success observed at the best practice site.

### Population and sampling

The study population comprised of pregnant women who newly registered for ANC services at 209 MDH supported health facilities in Dar es Salaam. At study baseline, quarter January–March 2015, these 209 health facilities registered 35,822 new pregnant women for ANC services; 4,588 at the six intervention sites and 31,234 at the 203 control sites.

### Description of the intervention

This intervention was designed based on initiatives that were implemented at the best practice site and showed remarkable success in improving male partner participation in ANC, couple HIV testing, and PMTCT. The core elements of the intervention were:

1. Sensitization, engagement and garnering commitment, from all clinical and non-clinical health facility staff and leadership, to provide couple friendly services
2. A facility-community integrated approach that encompassed both health facility and community-based interventions to improve male partner participation in ANC and PMTCT
3. Building a functional partnership between healthcare providers and local community leaders to collectively address male partner participation in ANC and PMTCT across health facility and community platforms
4. Identifying and engaging community leader champions who are informed and dedicated to lead initiatives for promotion of male partner participation in ANC and PMTCT at their communities.
5. Integrating male partner participation initiatives in existing routine community and health facility platforms e.g. community meetings and ANC health education sessions.
6. Redefining, packaging and delivering generalized key messages to the community focused on encouraging male partner participation in reproductive and child health services as a whole as opposed to ANC care or couple HIV testing/PMTCT services. Framing the messages this way specifically aimed at addressing barriers related to the pre-conceived notion that ANC clinics are for women only and the stigma associated with HIV-related services.

We hypothesized that a similar approach employed in other healthcare facilities would yield similar improvement in male partner participation. To test this hypothesis, we replicated the intervention at the six selected health facilities. We conducted a one-day baseline sensitization meeting with two healthcare providers from each of the six intervention sites. We shared the success story from the best practice site with the health care providers and the employed interventions. At the meeting, we also conducted focus group discussions with the providers to explore baseline couple HIV testing rates at their respective health facilities and develop action plans to improve it, building on successful initiatives from the best practice site. After returning to their health facilities, over a period of about one week, the two healthcare providers from each site, oriented their fellow providers and the health facility board. They also identified and oriented at least one community leader from their catchment area and partnered with them to implement the intervention. Together with the community leaders, providers from the intervention sites conducted a one-day visit to the best practice site to get practical exposure to the success story in male partner participation in PMTCT. At the best practice site, the healthcare providers and community leaders toured the facility to observe health education and couple HIV counseling and testing sessions, and conducted dialogues with community leaders leading the intervention, healthcare providers and couples who attended the ANC clinic. After the study tour, within a month’s time, facility specific actions plans were jointly reviewed, finalized and put to action by both healthcare providers and community leaders. We capitalized on the partnership, between healthcare providers and community leaders, to integrate male partner participation initiatives into community platforms thus maximizing their reach. Intervention sites were visited again six to nine months after the intervention for mentorship and supportive supervision to review progress of the intervention, identify and address challenges emerged. Control sites continued with routine standard of care which entailed basic ANC and PMTCT care with some non-specific/un-coordinated efforts to promote male partner participation, i.e. health education and priority services to couple, driven by health care providers.

### Data collection and analysis

Data was abstracted from the Tanzania national District Health Information System (DHIS-2), an online database managed by the Tanzania Ministry of Health Community Development, Gender, Elderly and Children (MOHCDGEC)—Health Management Information System (HMIS) unit [45]. This database records data from monthly health facility ANC summary reports submitted to the district health management office, by each health facility that provides ANC services in Tanzania. These reports aggregate basic socio-demographic, medical history, obstetric and ANC data, including HIV testing services, on all pregnant women that received ANC in each health facility each month. Data analysis was performed using Microsoft office excel software version 2011 and Stata version 15. Categorical variables were summarized using proportions to produce descriptive statistics for each site. Comparison between groups was assessed using tabi command and chi-square test in Stata version 15. The primary outcome of interest was the proportion of pregnant women who tested for HIV together with their partners during the first ANC visit (i.e. couple HIV testing). This was used as a proxy measure of male partner participation in ANC and PMTCT, as data to directly measure male partner attendance at ANC clinic was not routinely collected. Nevertheless, this was considered a good proxy measure of male partner ANC attendance as it was part of standard practice for all male partners accompanying their pregnant women to ANC clinic to be offered/ receive couple HIV counseling and testing.

### Sensitivity analysis

We performed two sensitivity analyses to assess if the effect of the intervention will persist under different scenarios, accounting for differences in the distribution of measured health facility characteristics across intervention and control sites. We used a regression model of the outcome “end line couple HIV testing rate” adjusting for baseline couple HIV testing rate. We first ran the regression model with all study sites, controlling for health facility ownership, level and volume of new pregnant women per day, as well as the baseline couple HIV testing rate. We then conducted an additional sensitivity analysis, limiting the control sites to 24, frequency matched 1:4 to the intervention sites by; health facility ownership, district location, level and volume of new pregnant women. In cases where there was an excess of matched control sites we used an alphabetical order to select the control site to include in the matching. Thereafter we re-ran the regression model with these 30 sites (6 intervention and 24 controls) using the outcome “end line couple HIV testing rate” and controlling for baseline couple HIV testing rate.

### Ethical clearance

This study was conducted as part of efforts to improve quality of routine healthcare services implemented by MDH in collaboration with Dar es Salaam regional and district health management offices. All data used for analysis was extracted from the existing de-identified aggregated health facility records available in the electronic DHIS2 database managed by MOHCDGEC-HMIS. Since the study involved secondary analysis of de-identified aggregate data, informed consent was not feasible, hence waiver of informed consent was sought, and obtained, along with the ethical clearance. Ethical clearance for analysis and publication of the data was provided by Muhimbili University of Health and Allied Sciences (Ref. No. 2017-06-28/AEC/Vol.XII/83) and the Tanzania National Institute for Medical Research (Ref. No. NIMR/HQ/R.8a/Vol. IX/2594) both in Dar es Salaam, Tanzania.

## Results

Table 1 describes characteristics of the intervention and control sites.

**Table 1:** Characteristics of pregnant women who registered for antenatal services at 209 health facilities in Dar es Salaam at baseline (January–March 2015), prior to the male partner intervention, and follow-up (January–March 2016).

Client Characteristics	Baseline January–March 2015		Follow-up January–March 2016	
	6 intervention sites (n = 4,588)	203 control sites (n = 31,234)	6 intervention sites (n = 4,260)	203 control sites (n = 30,950)
HIV testing	N (%)	N (%)	N (%)	N (%)
Tested for HIV with partner	528 (11.9)	4,869 (17.7)	1,463 (36.0)	5,414 (18.3)
Tested for HIV without partner	3,901 (88.1)	22,657 (82.3)	2,602 (64.0)	24,214 (81.7)
Age	N (%)	N (%)	N (%)	N (%)
< 20 years	565 (12.3)	4,334 (13.9)	658 (15.4)	3,648 (11.8)
≥20 years	4,023 (87.7)	26,900 (86.1)	3,602 (84.6)	27,302 (88.2)
Gestational age at first ANC^a^ visit	N (%)	N (%)	N (%)	N (%)
<12 weeks	506 (11.0)	4,856 (15.5)	428 (10.0)	3,416 (11.0)
≥12 weeks	4,082 (89.0)	26,378 (84.5)	3,832 (90.0)	27,534 (89.0)
HIV status at first ANC visit	N (%)	N (%)	N (%)	N (%)
Known HIV positive at entry	98 (2.2)	635 (2.3)	66 (1.6)	522 (1.7)
Tested HIV positive	197 (4.4)	1,147 (4.1)	151 (3.7)	940 (3.1)
Tested HIV negative	4,232 (93.4)	26,379 (93.6)	3,914 (94.7)	28,677 (95.2)

^a^ANC = Antenatal Care

At baseline in the first quarter (January–March) of 2015, a total of 4,588 and 31,234 pregnant women registered for ANC services at the six intervention and 203 control sites respectively (Table 2). The majority of the pregnant women were 20 years and older at both the intervention (87.7%) and the control sites (86.1%). Only 11.0% and 15.5% of the pregnant women at the intervention and the control sites, respectively, made their first ANC visit during the first trimester. At this visit, 4,429 (96.5%) and 27,526 (88.1%) women were counseled and tested for HIV, at the intervention versus the control sites, respectively. Among these women, 528 (11.9%) tested for HIV together with their partners at the intervention sites and 4,869 (17.7%) at the control sites at baseline.

**Table 2:** HIV testing rates at baseline (January—March 2015), prior to the male partner intervention, and at 1-year follow-up (January—March 2016) at 209 health facilities in Dar es Salaam, Tanzania.

	Baseline N (%)	Follow-up N (%)	p-value
Site A			
Tested for HIV with partner N (%)	105 (10.9)	108 (11.4)	0.77
Tested for HIV without partner N (%)	854 (89.1)	841 (88.6)	
Site B			
Tested for HIV with partner N (%)	20 (6.7)	77 (19.1)	<0.001
Tested for HIV without partner N (%)	280 (93.3)	327 (80.9)	
Site C			
Tested for HIV with partner N (%)	107 (9.3)	660 (74.6)	<0.001
Tested for HIV without partner N (%)	1,038 (90.7)	225 (25.4)	
Site D			
Tested for HIV with partner N (%)	194 (46.2)	414 (95.2)	<0.001
Tested for HIV without partner N (%)	226 (53.8)	21 (4.8)	
Site E			
Tested for HIV with partner N (%)	57 (4.7)	149 (15.1)	<0.001
Tested for HIV without partner N (%)	1,144 (95.3)	835 (84.9)	
Site F			
Tested for HIV with partner N (%)	45 (11.1)	55 (13.5)	0.3
Tested for HIV without partner N (%)	359 (88.9)	353 (86.5)	
All 6 intervention sites			
Tested for HIV with partner N (%)	528 (11.9)	1,463 (36.0)	<0.001
Tested for HIV without partner N (%)	3,901 (88.1)	2,602 (64.0)	
All 203 control sites			
Tested for HIV with partner N (%)	4,869 (17.7)	5,414 (18.3)	0.07
Tested for HIV without partner N (%)	22,657 (82.3)	24,203 (81.7)	
103 public control sites			
Tested for HIV with partner N (%)	4,010 (17.9)	4,406 (18.6)	0.10
Tested for HIV without partner N (%)	18,331 (82.1)	19,344 (81.4)	
170 health center/ dispensary control sites			
Tested for HIV with partner N (%)	3,732 (17.6)	4,221 (19.1)	<0.001
Tested for HIV without partner N (%)	17,465 (82.4)	17,829 (80.9)	
109 control sites attending at least 1 new pregnant woman per day			
Tested for HIV with partner N (%)	4,159 (16.5)	4,409 (16.9)	0.22
Tested for HIV without partner N (%)	20,981 (83.5)	21,604 (83.1)	
24 matched control sites			
Tested for HIV with partner N (%)	1,351 (16.0)	1,380 (16.7)	0.22
Tested for HIV without partner N (%)	7,080 (84.0)	6,871 (83.3)	

**Fig 1:**
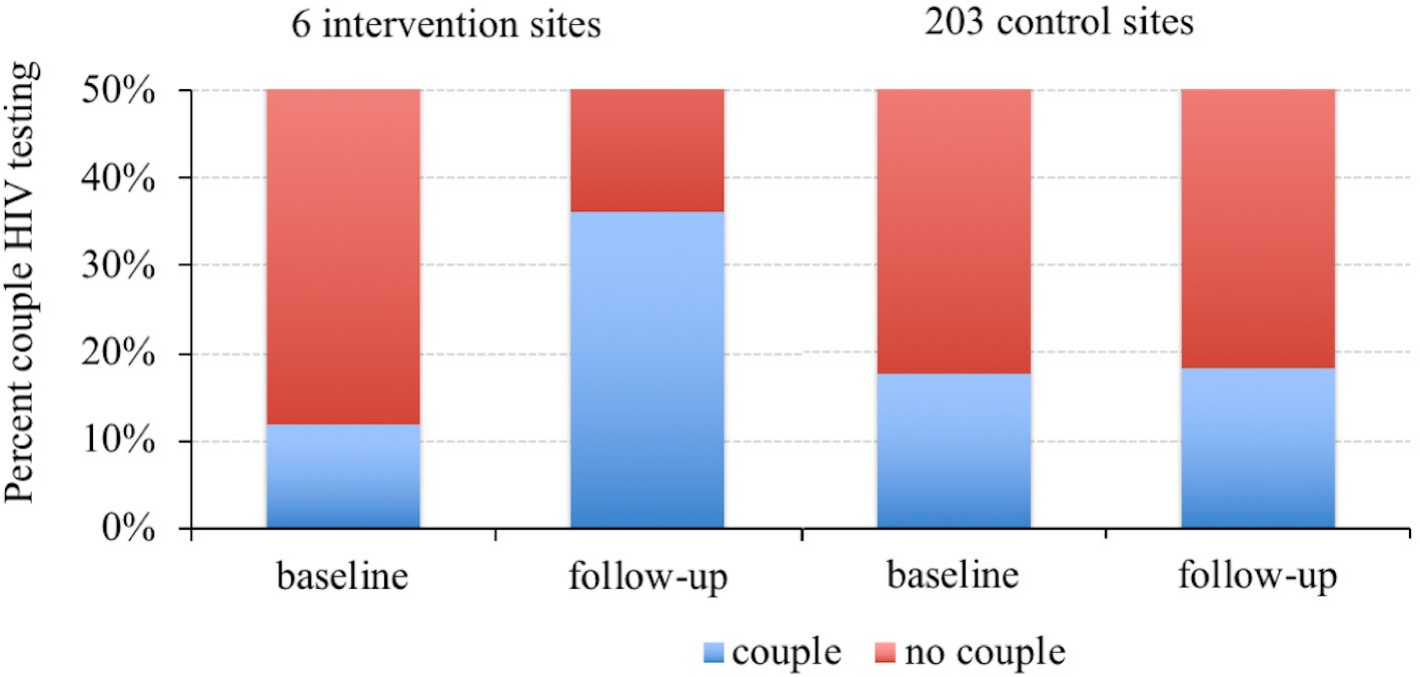
Couple HIV testing rates among pregnant women registering for antenatal services at 209 health facilities in Dar es Salaam, Tanzania.

At one-year follow-up, in the first quarter (January-March) of 2016, the overall couple HIV testing tripled from 11.9% to 36.0% (p <0.001) at the six intervention sites whereas it remained relatively unchanged (from 17.7% to 18.3%, p = 0.07) at the 203 control sites (Fig 1). During this period 4,065 (95.4%) pregnant women were tested for HIV at their first ANC visit in the intervention sites and 29,628 (95.8%) in the control sites. As observed at baseline, the majority of the women were 20 years and older in both the intervention (84.6%) and control (88.2%) sites and only 10.0% and 11.0% at the intervention and control sites, respectively, were in their first trimester.

**Fig 2:**
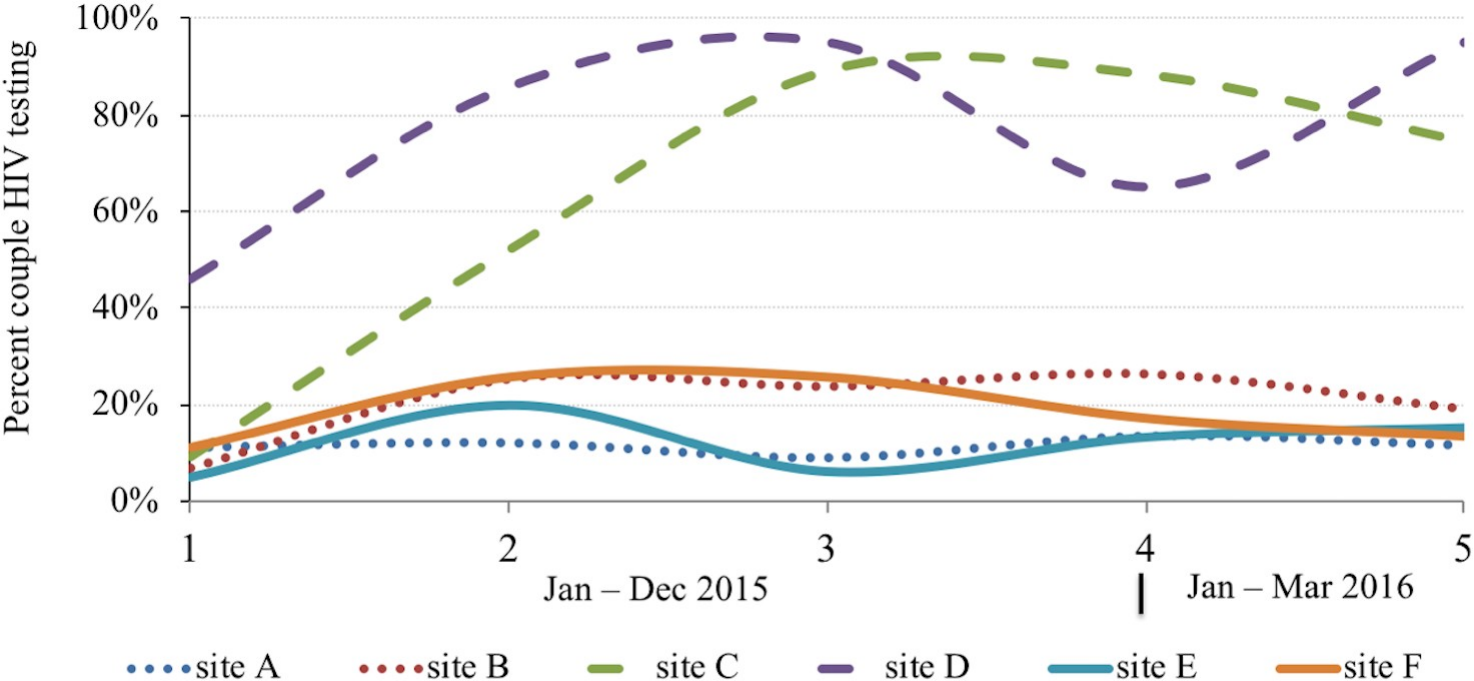
Couple HIV testing rates across quarters at first antenatal care visit at each of the six intervention sites from 1^st^ January– 31^st^ March 2015 to 2016.

We observed wide variation in couple HIV testing rates across sites A to E throughout the intervention (Fig 2). Four intervention sites (B, C, D and E) achieved statistically significant improvements in couple HIV testing at one-year follow-up compared to baseline (19.1% from 6.7%, p <0.001; 74.6% from 9.3%, p<0.001; 95.2% from 46.2%, p<0.001 and 15.1% from 4.7%, p<0.001). These improvements were equivalent to two- to eight-fold increases in couple HIV testing rates at follow-up relative to baseline rates (ratio of proportions = 2.9; 8.0; 2.1; and 3.2, p<0.001 across all four sites). The remaining two intervention sites A and F observed non-significant changes in couple HIV testing at follow-up compared to baseline (from 10.9% to 11.4%, p = 0.8 and 11.1% to 13.5%, p = 0.3).

We conducted mentorship and supportive supervision visits to all the intervention sites to discuss with healthcare providers about their experience implementing the intervention and provide support as needed. During these visits, we discovered that the level of engagement, commitment and partnership between community leaders and healthcare providers, and their active participation in implementing the intervention varied across sites. Four sites C, D, E and F reported continued engagement and functional partnership with community leaders throughout the intervention period. Sites A and B, in Ilala district, struggled to get and/or maintain support from their community leaders. Sites E and F located in Temeke district reported to have had a particularly challenging experience getting a positive response and support from the community in general and male partners. According to the healthcare providers at site E and F, a major barrier that limited their success was the strong belief of the community they serve with regard to separation of gender roles among men and women. These providers further remarked that their communities were more culturally accustomed to ANC services being destined for women only, therefore, viewing attendance and participation of men as inappropriate.

**Fig 3:**
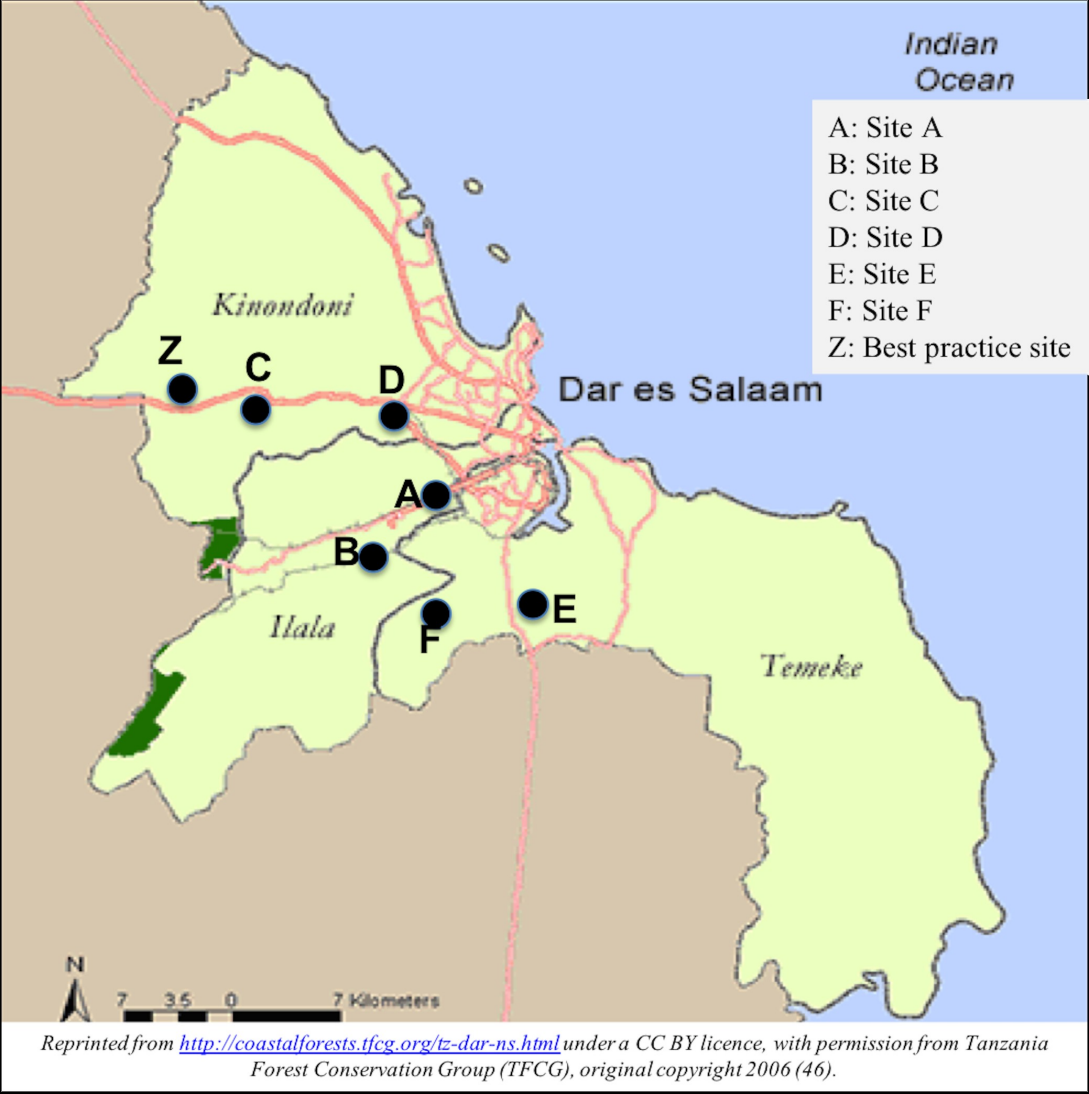
Map of Dar es Salaam showing location of the six intervention sites in relation to the best practice site. Reprinted from http://coastalforests.tfeg.org/tz-dar0ns.html under a CC BY license, with permission from Tanzania Forest Conservation Group (TFCG), original copyright 2006 [46].

Of note, the two intervention sites C and D that achieved remarkably high couple HIV testing rates of 95.2% and 74.6% were located in the same administrative district (Kinondoni) and relatively closer to the best practice site Z (Fig 3). These sites share a major road connecting them to the best practice site as illustrated in Fig 3.

We conducted sensitivity analyses, using a regression model of the end line couple HIV testing rate controlling for baseline couple HIV testing rate, to assess stability of our findings in different scenarios. This analysis revealed consistent but non-statistically significant results–indicating a tendency towards higher couple HIV testing rate in intervention than control sites—adjusting for differences in health facility ownership, level, volume of new pregnant women, and baseline couple HIV testing rate (β-coefficient [95%CI] (23.4 [-0.9, 47.8], p = 0.06). Further sensitivity analysis limiting control sites to 24 health facilities, frequency matched 1:4 to intervention sites by health facility ownership, district location, level and volume of new pregnant women, revealed similar results; β-coefficient [95%CI] (21.6 [-1.0, 44.3], p = 0.06).

## Discussion

Our study evaluated whether community leader engagement can improve male partner participation in ANC and PMTCT services. We measured male partner participation using the proportion of pregnant women who tested for HIV together with their partners (couple HIV testing) at their first ANC visit. Our findings show that the six intervention sites that engaged community leaders tripled couple HIV testing to 36.0% from 11.9%, p<0.001, whereas there was no significant change (18.3% from 17.7%, p = 0.07) in the 203 control sites. Sensitivity analyses by health facility ownership, district location, level and volume of new pregnant women revealed consistent but non-statistically significant results, supporting a tendency towards higher couple HIV testing rates in intervention than control sites.

Our findings shed light on the potential of a simple, inexpensive and easily scalable intervention—implemented over a period of one year—to effect meaningful change in the way couples and the community perceive and access ANC services. Several other studies have investigated different approaches to improve male partner participation in ANC and PMTCT. In a study done in southern Tanzania, 31% of pregnant women who were given invitation letters to give to their partners, compared to 28% of the women instructed to verbally invite their partners, returned with their partners at the subsequent ANC visit [36]. Similarly, a study in Malawi showed that 28% of pregnant women who received a written invitation came back with partners compared to 19% who received verbal invitation [37]. In a South African study, 35% of pregnant women who received written invitation letters came back with their partners at the subsequent ANC visit [38]. Emphasizing on the role of peers, another study in Malawi found that the use of peer couples in health education and drama at the health facility and creating a male friendly environment improved male partner participation at ANC from 1% to 11% [39]. Similarly a study done by Audet et al in Mozambique, showed that engagement of trained male peers improved male partner ANC attendance from 5% to 34% [20]. Our findings add to this larger body of literature that reaffirms the feasibility of improving male partner participation in ANC, couple HIV counseling and testing, and PMTCT services. Engagement of community leaders to advocate for effective utilization of ANC and PMTCT services, which we employed in our study, is a unique and innovative approach that leverages the inherent role and influence of community leaders to contribute towards health endeavors. Community leaders are particularly suited to address low male partner participation in ANC and PMTCT services as they have direct access to the community and the potential to influence male partner participation from the first ANC visit, which is most important in PMTCT care.

An interesting finding in our study is the widely varying rates of couple HIV testing at follow-up in the six intervention sites. We observed improvements in couple HIV testing at follow-up in four of the intervention sites ranging from two- to eight-fold increases relative to the baseline rates (ratio of proportions = 2.9; 8.0; 2.1; and 3.2, p<0.001 across all four sites), whereas there was no significant change in the other two sites. These differences may be due to the different levels of engagement, acceptance and participation of healthcare providers and community leaders in the intervention. Based on the experience and success of the best practice site, a major pre-requisite for success of the intervention relies on successful engagement, acceptance, ownership and active participation of community leaders and the community at large. As observed during the supportive supervision and mentorship, four intervention sites that succeeded to mobilize engagement and active participation of community leaders, observed significant improvement in couple HIV testing rates. Unsurprisingly two of the intervention sites, located within the same administrative district and geographically closer to the best practice site, achieved remarkably high couple HIV testing rates of 95.2% and 74.6%. This result may have been influenced by the overlap of the community served by these sites with the best practice site. This overlap suggests that communities served by these two sites may have been exposed to the intervention much earlier, due to the spillover effects from the best practice site and catchment area, as suggested by the higher (46.2%) baseline couple HIV testing rate in site D. Furthermore, community leaders from these two sites had greater access to and interaction with their counterparts from the best practice site due to sharing of administrative district (i.e. through routine administrative platforms and activities at the district). This is likely to have contributed to greater engagement and acceptance of the intervention by the community leaders at these 2 intervention sites. This physical proximity and overlap of communities served may have pre-empted the community leaders, and community in general, to be more receptive to the interventions. Conversely, failure to observe improvement at follow-up in two sites (A and F) highlights the critical role of persistent and sustained engagement of community leaders and the community, which is needed to realize meaningful change in male involvement in ANC and PMTCT. Whereas site F observed some improvement across quarters (i.e. from 11% at baseline-Q1-2015 to 26% in Q2, 26% in Q3, 17% in Q4, and 13% in Q1-2016), progress across quarters in Site A was relatively flat (i.e. from 11% at baseline to 12% in Q2, 9% in Q3, 13% in Q4 and 11% in Q1-2016). The encountered difficulties in securing acceptance of the intervention from the community (Site F), and in particular, community leaders (Site A) were likely major reasons for the failure to observe improvement. This observation underscores the importance of effective engagement, securing support and active participation of community leaders to achieve higher and sustained success rates.

Our study suggests that local community leaders, in partnership with healthcare providers, have the potential to influence the community’s attitude and behavior towards couple ANC attendance and HIV testing at ANC clinic. In Tanzania and most of sub-Saharan Africa, community leaders enjoy respect and influence from both men and women in the community they serve. This makes them uniquely positioned to address barriers to male partner participation in ANC and PMTCT. These barriers, such as: gender-role stereotypes, inadequate knowledge, fear of knowing HIV status and HIV-related stigma [29, 30, 35], are rooted in the community’s awareness, attitude and behavior. Therefore, to stimulate change in behavior and attitude, we sensitized, secured support and engaged community leaders to lead initiatives to promote male partner participation in ANC and PMTCT. This enabled us to harness the respect and influence they have in community and direct it towards a health cause. Community leaders also have unlimited access to community platforms used to address various issues of interest to the community. By partnering with community leaders, we gained access to these important community platforms and achieved successful integration of initiatives to advocate for male partner participation in ANC and PMTCT. Through these platforms, community leaders mobilized community support and passed resolutions that adopted an opt-out approach for male partner participation in ANC. This approach advocated for all pregnant women to attend the first ANC visit with their partners. Women who could not attend the clinic together with their partners were still offered care, as required by national policy and guidelines, but also counseled on the importance of male partner attendance and referred to their community leaders for further dialogue, intervention and support.

Potential limitations of our study include the purposive selection of few intervention sites with favorable conditions to support a successful intervention, which may have resulted in biased comparisons and limited generalizability of study findings. Integrating the intervention in routine care settings may also have made the intervention and control sites subjective to other un-measured influences such as co-interventions, diffusion of interventions across sites. Use of aggregated data also limited our ability to robustly analyze the data to determine individual-level effects of the characteristics of study participants on the outcomes including disparities in age distribution, socio-economic and health status of women and families in intervention and control sites. We have—however—included in table one, available information on the distribution of broad age categories and timing of ANC visit across intervention and control sites.

Other limitations include the fact that this study did not collect information on untoward effects of the intervention such as refusal of care because of failure to attend ANC clinic with partner, deterrence of ANC clinic attendance as a result of the intervention, or any form of abuse to the woman as a result of requesting the partner to accompany her to the ANC clinic. This limits our ability to assess adverse effects of the intervention and, by reducing the denominator of couple HIV testing, may have biased the results in favor of the intervention. Available data indicates that, at follow-up, there were 7.1% fewer women who newly registered for ANC services at the six intervention sites compared to 0.9% at 203 control sites. Furthermore, we did not collect any information to verify whether partners who attended the ANC clinic were true sexual partners of the index pregnant women. It has been previously reported that women in similar situations may sometimes engage or pay other non-sexual partners to accompany them to the ANC clinic in order to satisfy this requirement or avoid refusal of care [47]. This may result in incorrect interpretation of positive study findings whereas actual intended positive health outcomes i.e. male sexual partner engagement in ANC, couple HIV counseling and testing, and PMTCT–have not improved. Nevertheless we believe that these negative effects and practices were less likely because our intervention primarily aimed at the community at large as opposed to unilateral focus on pregnant women who were not accompanied by their partners during ANC. The intervention focused on changing mindset and attitude of the community with regard to the role and active participation of men in reproductive health, ANC and PMTCT services; however, long-term effects remain to be studied.

Overall, our study shows that effective engagement of community leaders was followed by rapid improvement in male partner participation in ANC and PMTCT services.

## Conclusions

Overall, our study suggests that engagement of community leaders–a simple, in-expensive and scalable intervention–can contribute to addressing barriers to male partner participation in ANC and PMTCT. The intervention’s focus at the community level positions it well to address societal-level barriers and stimulate change in attitude and behavior towards male partner participation in ANC and PMTCT. By strengthening male partner participation, the intervention can contribute towards improving uptake, adherence and retention in ANC and PMTCT services among pregnant women, their partners, infants and families. This, therefore, ensures effective delivery of PMTCT interventions so as to have maximum impact in eliminating new HIV-infections in children born to women living with HIV. We recommend further studies, with random assignment of intervention and control sites, and different contextual conditions, to replicate this approach and reaffirm the effectiveness and scalability of this intervention.

## Supporting information

S1 File Permission to publish image in Fig 3. (PDF)
