# Supplementary material for: Engaging community leaders to improve male partner participation in the prevention of mother-to-child transmission of HIV in Dar es Salaam, Tanzania
Source: PLoS One. 2018 Dec 12;13(12):e0207986. doi: 10.1371/journal.pone.0207986 (PMC6291081; doi:10.1371/journal.pone.0207986)
Supplement: S1 File — (PDF) [file pone.0207986.s001.pdf]

Subject: Re: request permission to use a graphical image on your website
Date: Wednesday, August 1, 2018 at 7:14:58 PM East Africa Time
From: ndoggart@tfcg.or.tz
To: Goodluck Lyatuu
CC: tfcg@tfcg.or.tz, Charles Kilewo, Anna Mia Ekström, Gunnel Biberfeld, Helga

Dear Goodluck

I confirm that you have our permission to publish the map under the creative commons license.

Kind regards
Nike

Nike Doggart
Senior Technical Advisor
Tanzania Forest Conservation Group
www.tfcg.org

On 2018-08-01 05:34, Goodluck Lyatuu wrote:
Dear Ms. Doggart
I trust that you are well
I am writing to follow up on my email below
Thank you in advance
Regards
Goodluck
On 7/24/18, 7:07 AM, "Goodluck Lyatuu" <goodluck.lyatuu@ki.se> wrote:
Dear Ms. Nike Doggart
I trust that you are well
I am writing in reference to my earlier granted permission (17 March 2018, see below) to use a modified version of a map of Dar es Salaam region posted on your website: http://coastalforests.tfcg.org/tz-dar-ns.html , in a manuscript that was submitted to PLOS ONE.
First, I wish to express my sincere appreciation for the permission you provided earlier, which allowed me to submit the manuscript to PLOS ONE on 16th March 2018
Upon feedback and instructions from journal reviewers and editor, I kindly further request for a written permission for the open-access journal PLOS ONE to publish a modified version of the map of Dar es Salaam region found on your website (http://coastalforests.tfcg.org/tz-dar-ns.html) under the Creative Commons Attribution License (CCAL) CC BY 4.0 (http://creativecommons.org/licenses/by/4.0/) Please be aware that this license allows unrestricted use and distribution, even commercially, by third parties. Please reply and provide explicit written permission to publish the aforementioned map under a CC BY license.
Kindly advise if there is any further information needed
Thank you in advance
Regards
Goodluck
On 3/17/18, 8:52 PM, "ndoggart@tfcg.or.tz" <ndoggart@tfcg.or.tz> wrote:
Dear Dr Lyatuu
Thank you for requesting TFCG's permission to use the map.

On behalf of TFCG, I confirm that we have no objections to your
using
the map for your publication.
I wish you every success with your research.
Kind regards
Nike
Nike Doggart
Senior Technical Advisor
Tanzania Forest Conservation Group
On 2018-03-17 19:53, Goodluck Lyatuu wrote:
> Dear TFCG
>
> Hello
>
> I am writing to request permission to use a modified version
of the
> map posted on your website in my research article titled,
“Engaging
> community leaders to improve male partner participation in
the
> prevention of mother-to-child transmission of HIV in Dar es
Salaam,
> Tanzania” that I wish to submit for scientific publication at
the
> PLOSONE. Here is the link to the map on your website:
> http://coastalforests.tfcg.org/tz-dar-ns.html
>
> Also attached is the modified version of the map for your
review and
> approval.
>
> The research article in question presents findings of a
biomedical
> implementation research - conducted in Dar es Salaam,
Tanzania –
> that aimed at investigating the role of community leaders in
improving
> male-partner participation in; antenatal care clinic
(accompanying
> their pregnant women), couple HIV counseling and testing, and
> prevention of mother-to-child transmission of HIV (PMTCT)
services.
>
> I am a medical doctor and public health specialist working at
> Management and Development for Health (MDH) [www.mdh-tz.org
[1]] , a
> public health non-governmental organization dedicated towards
> addressing public health priorities in Tanzania and beyond. I
am also,
> currently, enrolled in PhD studies at Muhimbili University of
Health
> and Allied Sciences (MUHAS) in Dar es Salaam and Karolinska
> Institutet, Stockholm, Sweden.
>
> Thank you in advance and looking forward to hear from you
soon
>
> Also copied are my academic supervisors

>
> Regards
>
> Goodluck Lyatuu
>
> Management and Development for Health
>
>
>
> Links:
> ------
> [1] http://www.mdh-tz.org
